# Multiplex PCR-based rapid pathogen identification in acute cholecystitis using the FilmArray BCID2 panel

**DOI:** 10.3389/fmicb.2025.1687205

**Published:** 2025-10-30

**Authors:** Gaku Nakamura, Koji Asai, Ryutaro Watanabe, Makoto Kuroda, Yu Shibahara, Shintaro Teraoka, Osahiko Hagiwara, Nanako Kakizaki, Jiro Sato, Manabu Watanabe, Yoshihisa Saida

**Affiliations:** ^1^Department of Surgery, Toho University Ohashi Medical Center, Tokyo, Japan; ^2^Department of Clinical Oncology, Toho University Graduate School of Medicine, Tokyo, Japan; ^3^Department of Medical Laboratory Science, Faculty of Health Science, Kumamoto Health Science University, Kumamoto, Japan

**Keywords:** acute cholecystitis, acute biliary infection, multiplex PCR system, antimicrobial resistance, blood culture identification 2

## Abstract

**Purpose:**

Acute biliary infections are serious conditions in which delayed or inappropriate therapy can worsen clinical outcomes and promote antimicrobial resistance. This study evaluated the FilmArray® Blood Culture Identification 2 (BCID2) panel, a multiplex PCR system, for the rapid detection of pathogens in bile samples from patients with acute cholecystitis.

**Methods:**

Bile samples were collected intraoperatively or by percutaneous aspiration from 77 consecutive patients with acute cholecystitis. Each specimen was divided for conventional culture and BCID2 panel testing. Culture results served as the reference standard to calculate positive and negative percent agreement (PPA and NPA), predictive values, and accuracy.

**Results:**

Bacterial growth was observed in 34 cases (44.2%), yielding 57 isolates from 16 species. The BCID2 panel correctly detected 41 of 46 BCID2-targetable isolates. For detecting at least one pathogen, PPA (sensitivity) was 82.4%, NPA (specificity) 100%, positive predictive value 100%, negative predictive value 91.5%, and overall accuracy 92.2%. Detection was achieved in 70.6% of monomicrobial and 94.1% of polymicrobial infections, with higher bacterial loads (>10^6^ CFU/mL) associated with improved detection rates.

**Conclusion:**

Multiplex PCR testing using BCID2 panel enables rapid and accurate identification of causative bacteria directly from bile. This approach may support earlier targeted therapy and promote appropriate antimicrobial stewardship in acute biliary infections.

## 1 Introduction

Acute biliary infections, including acute cholecystitis and cholangitis, are inflammatory diseases of the gallbladder and bile ducts that are associated with high morbidity and mortality (Gomi et al., 2018). Timely and appropriate intervention is crucial for improving patient outcomes, as delays or inappropriate therapy can lead to severe disease progression (Gomi et al., 2018; Sartelli et al., 2024). Inadequate initial antimicrobial treatment may further exacerbate the condition, and the increasing prevalence of antimicrobial-resistant bacteria—particularly among Enterobacterales—has become a global healthcare concern (Goo et al., 2012; Jean et al., 2014; Coccolini et al., 2015; Liu et al., 2020). Therefore, establishing rapid and accurate diagnostic methods to identify causative pathogens is critically important for improving diagnostic efficiency and supporting appropriate antimicrobial selection.

The Tokyo Guidelines 2018 (TG18) recommend performing bile culture and antibiotic susceptibility testing to identify causative organisms in acute biliary infections (Gomi et al., 2018). While targeted antimicrobial therapy based on culture results is essential, conventional bile culture requires approximately 5 days to yield results (Kujiraoka et al., 2017; Watanabe et al., 2021; Yoon et al., 2022). This delay necessitates empirical broad-spectrum antibiotic use, which can contribute to the emergence of multidrug-resistant organisms (Gomi et al., 2018; Sartelli et al., 2024). Indeed, antimicrobial-resistant organisms are a worldwide healthcare challenge, and the introduction of rapid diagnostic technologies is considered key to curbing the spread of resistance (Coccolini et al., 2015; Sartelli et al., 2024).

We previously conducted several investigations into the rapid identification of causative organisms in acute biliary infections. As an initial approach, we performed an extensive metagenomic assessment of bile from patients with acute biliary infection (Kujiraoka et al., 2017). This method enabled bacterial identification within 24 h, demonstrating the feasibility of broad-range pathogen detection. However, metagenomic testing was constrained by high costs and labor-intensive protocols. Subsequently, we assessed an automated multigene detection platform (Verigene®, Nanosphere Inc., Northbrook, IL, USA) for pathogen identification in bile samples obtained from patients with acute cholecystitis (Watanabe et al., 2021), and this system identified bacterial species and resistance genes within approximately 2 h. Nevertheless, the detection rate with Verigene was lower than anticipated (~35.7%), which may be attributed to low bacterial loads or the presence of multiple bacterial species in some samples.

Recently, multiplex PCR systems have attracted increasing attention for their ability to simultaneously detect multiple pathogens and resistance genes, delivering results within approximately 1 h (Micó et al., 2015; Ciesielczuk et al., 2018; Lotte et al., 2022). This approach dramatically shortens the time to diagnosis compared to traditional cultures, with high sensitivity and specificity demonstrated in various clinical settings. However, no commercially available panels are specifically designed for acute abdominal infections or acute biliary infections. To address this gap, in our previous study we employed the Blood Culture Identification (BCID) panel, which was the initial version of this system, and applied it off-label to 10 cases of intra-abdominal infection, including two bile specimens, performing metagenomic analysis for in-depth characterization of the bacterial communities (Kakizaki et al., 2023). This study demonstrated that BCID panel not only achieved high sensitivity but also successfully identified the bacterial species in bile samples, highlighting its potential applicability to acute biliary infections.

Since then, the FilmArray® Blood Culture Identification 2 (BCID2) panel has become available, providing expanded coverage of clinically relevant pathogens and a broader range of resistance genes, particularly among Enterobacterales. Given the recent increase in antimicrobial resistance among Enterobacterales isolated from patients with acute biliary infection, the BCID2 panel may offer significant advantages in this clinical context. Although in our previous study bile samples were limited to only two cases, several investigations have assessed multiplex PCR in ascites or pus from intra-abdominal infections. For example, in spontaneous bacterial peritonitis, direct identification using multiplex PCR significantly reduced the time to optimal antibiotic therapy (Lotte et al., 2022). Similarly, in intra-abdominal infections, multiplex PCR detected additional anaerobes missed by culture and reduced time to results by over 17 h (Ciesielczuk et al., 2018). These findings indicate that the advantages of multiplex PCR observed in other intra-abdominal infections may also extend to the management of acute biliary infections. Therefore, the aim of the present study was to evaluate the effectiveness of the BCID2 panel in the rapid identification of causative organisms in acute biliary infections.

## 2 Methods

### 2.1 Patients

This study included 77 consecutive patients who underwent surgery for acute cholecystitis at Toho University Ohashi Medical Center between February 2022 and January 2024. All patients underwent diagnostic evaluation for initial symptoms such as abdominal pain, nausea, and fever, and acute cholecystitis was confirmed in accordance with the TG18 (Yokoe et al., 2018). Disease severity was subsequently assessed (Yokoe et al., 2018), and surgical fitness was evaluated following the TG18 criteria (Okamoto et al., 2018). At our institution, early laparoscopic cholecystectomy is the standard treatment policy for acute cholecystitis (Asai et al., 2017). In this cohort of consecutive patients analyzed, all cases were community-acquired acute cholecystitis, and no healthcare-associated infections were observed. The study protocol was approved by the institutional ethics committee of our university hospital (approval no. H23029_H21090_H17077) as well as the ethics committee of the National Institute of Infectious Diseases (approval no. 1588). Written informed consent was obtained from all patients prior to specimen analysis.

### 2.2 Bile sample collection

Bile samples were obtained from each patient under sterile conditions. In 74 of the 77 patients (96.1%), bile was collected intraoperatively during cholecystectomy. In the remaining cases, bile samples were obtained by percutaneous transhepatic gallbladder aspiration preoperatively. Each specimen (approximately 2–3 mL) was divided equally into two anaerobic transport vials. One vial was submitted to the hospital microbiology laboratory for culture and antimicrobial susceptibility testing, and the other was immediately frozen at −20 °C for analysis by the multiplex PCR system. This study was based exclusively on clinical bile specimens obtained from patients with acute cholecystitis, and ATCC reference strains were not included.

### 2.3 Antimicrobial susceptibility testing

Bile specimens were inoculated onto both aerobic and anaerobic culture media and cultured according to standard microbiological procedures, using 5% sheep blood agar as a non-selective medium and deoxycholate hydrogen sulfide lactose agar as a selective medium. Bacterial species identification was performed based on biochemical characteristics. Antimicrobial susceptibility testing of cultured isolates was conducted using the DxM Microscan WalkAway system (Beckman Coulter, Brea, CA, USA) with the Neg Combo EN 2 T® panel, the Neg Conbo NF 3 T® panel, and the Pos Combo 2 T® panel. The Neg EN Combo 1 T® panel includes 19 antimicrobial agents: Ampicillin, Ampicillin/Sulbactam, Cefazolin, Ceftazidime, Cefepime, Piperacillin/Tazobactam, Aztreonam, Meropenem, Gentamicin, Amikacin, Levofloxacin, Sulfamethoxazole/Trimethoprim, Cefmetazole sodium, Cefpodoxime, Cefpodoxime/Clavulanic acid, Ceftriaxone, Latamoxef sodium, and Nalidixic acid. The Neg Combo NF3T® panel includes 14 antimicrobial agents: Piperacillin/Tazobactam, Ceftazidime, Cefepime, Meropenem, Imipenem, Aztreonam, Gentamicin, Amikacin, Ampicillin/Sulbactam, Ciprofloxacin, Colistin, Minocycline, Piperacillin/Tazobactam, and Sulfamethoxazole/Trimethoprim. The Pos Combo 2 T® panel includes 19 antimicrobial agents: Ampicillin, Arbekacin sulfate, Cefazolin, Cefoxitin, Clindamycin, Daptomycin, Erythromycin, Gentamicin, Imipenem, Erythromycin / Clindamycin, Levofloxacin, Linezolid, Minocycline, Oxacillin, Penicillin, Rifampicin, Teicoplanin, Sulfamethoxazole/Trimethoprim, and Vancomycin. All results were interpreted according to the Clinical and Laboratory Standards Institute (CLSI) guidelines (document M100-S26). Detailed antimicrobial susceptibility testing results of all bacterial isolates are presented in Supplementary Table 1.

### 2.4 Multiplex PCR system assays

We used a commercially available multiplex real-time PCR system (FilmArray® Blood Culture Identification 2: BCID 2 panel, BioFire Diagnostics, Salt Lake City, UT, USA). Although validated for positive blood culture bottles, the BCID2 panel was applied off-label to bile specimens in this study to evaluate its diagnostic utility. For each sample, bile was frozen immediately after collection, thawed before analysis, and directly loaded into the FilmArray® cartridge without additional pre-analytical processing, according to the manufacturer’s instructions.

### 2.5 Definitions used for the comparison of multiplex PCR system assay results with bacterial culture findings

The performance of the BCID2 panel was evaluated by comparison with culture results. Positive percent agreement (PPA) was defined as the proportion of culture-positive cases that were also positive by PCR, and negative percent agreement (NPA) as the proportion of culture-negative cases that were also negative by PCR. These parameters are equivalent to sensitivity and specificity, respectively, and positive predictive value (PPV), and negative predictive value (NPV) were calculated using standard 2 × 2 contingency tables, as described previously (Buchan et al., 2020; Caméléna et al., 2023).

### 2.6 Statistical analysis

For categorical data, we used Fisher’s exact test. Continuous variables were compared using the Mann–Whitney U test. Continuous data are presented as median (range). A two-tailed *p*-value < 0.05 was considered statistically significant. All statistical analyses were performed using EZR version 1.68 on Windows.

## 3 Result

Patient characteristics are shown in Table 1. The median age of the patients was 66 years (range 22–94), with 53 men (68.8%) and 24 women (31.2%). Abdominal pain was present in 72 patients (93.5%), and nausea was observed in 16 patients (20.8%). The median body temperature was 36.7 °C (range, 35.4–38.3 °C), the white blood cell (WBC) count was 10.2 × 10^3^/μL (range, 2.9–21.9 × 10^3^/μL), and the C-reactive protein (CRP) level was 8.19 mg/dL (range, 0.14–43.51 mg/dL). Imaging findings characteristic of acute cholecystitis, such as gallbladder distension and wall thickening, were observed in all patients. No postoperative infectious complications were observed, and the median postoperative hospital stay was 5 days (range, 3–59 days).

**Table 1:** Patient characteristics.

Factor	*n* = 77	Factor	*n* = 77
Sex (M/F)	53/24	Age (years)	66 (22–94)
Abdominal pain (Y/N)	72 / 5	Nausea (Y/N)	16/61
ASA-PS	2 (1–4)	CCI	4 (0–12)
Body Temperature (°C)	36.7 (35.4–38.3)	WBC (×10^3^/μL)	10.2 (2.9–21.9)
CRP (mg/dL)	8.19 (0.14–43.51)	T-Bil (mg/dL)	1.2 (0.2–7.4)
Bile culture (Positive/Negative)	34/43		

ASA-PS: American society of anesthesiologists - physical status; CCI: Charlson comorbidity index, WBC: White blood cell, CRP: C-reactive protein.

### 3.1 Bacterial isolations and multiplex PCR detection

Bile cultures were positive in 34 of the 77 samples (44.2%), yielding a total of 57 bacterial isolates representing 16 different species (Table 2). The most frequently isolated organisms were *E. coli* (16 isolates, including 2 extended-spectrum beta-lactamase [ESBL]-producing strains; 28.1%), *Streptococcus* spp. (10 isolates, 17.5%), and *Klebsiella pneumoniae* (6 isolates, 10.5%).

**Table 2:** Bacterial species isolated from bile cultures, classified by inclusion in the BCID2 panel.

Including in the BCID2 panel		Not including in the BCID2 panel	
Microorganism	*n*	Microorganism	*n*
Gram-negative bacteria		Gram-negative bacteria	
*E. coli*	16	*Aeromonas* spp.	2
*Klebsiella pneumoniae*	6		
*Klebsiella oxytoca*	2		
*Pseudomonas aeruginosa*	1		
*Enterobacter aerogenes*	1		
Gram-positive bacteria		Gram-positive bacteria	
*Streptococcus* spp.	10	*Enterococcus casseliflavus*	2
*Staphylococcus aureus*	3	*Corynebacterium* sp.	1
*Enterococcus* spp.	3		
*Enterococcus faecium*	2		
*Enterococcus faecalis*	2		
Anaerobic bacteria		Anaerobic bacteria	
		*Clostridium perfringens*	2
		*Bacteroides vulgatus*	2
		*Anaerobic* GNR*	2
Total	46	Total	11

*GNR: Gram-negative rods.

All 77 bile samples were successfully analyzed by the BCID2 panel without the need for prior culture or any software errors. Out of the 57 cultured isolates, 46 (80.7%) species were included in the BCID2 panel’s detection range. The BCID2 panel correctly identified 41 of these 46 isolates, yielding a detection sensitivity of 89.1% for organisms covered by the panel. In addition, ESBL-producing *E. coli* were identified in two patients, and both isolates were appropriately detected as CTX-M positive by the BCID2 panel. In addition, 11 isolates of species not included in the BCID2 panel were obtained by culture, including anaerobes (e.g., *Bacteroides vulgatus*, *Clostridium perfringens*), *Aeromonas* spp., and *Enterococcus* spp., which, by definition, were outside the detection scope of the BCID2 panel.

Of the 46 bacterial species detectable by the BCID2 panel, 7 species (15.2%) were not identified (false negative), including *Streptococcus* spp. (*n* = 4), *Klebsiella pneumoniae* (*n* = 1), *Enterococcus* spp. (*n* = 1), and *Enterococcus faecalis* (*n* = 1) (Table 3). On the other hand, there were 10 bacterial isolates that were judged as positive by BCID 2 panel despite not being confirmed by bacterial culture (false positives). These included *Enterococcus faecium* (*n* = 4), *Klebsiella pneumoniae* (*n* = 3), *Klebsiella oxytoca* (*n* = 1), *Proteus* spp. (*n* = 1), *Bacteroides fragilis* (*n* = 1), and CTX-M–producing Enterobacterales (*n* = 1).

**Table 3:** Case-by-case comparison of BCID2 panel and culture results for false- negative and false- positive cases.

False-negative	Cultures	BCID2
Case 1	***Klebsiella pneumoniae***	Negative
Case 2	***Streptococcus* spp.**	Negative
Case 3	***Streptococcus* spp.**	Negative
Case 4	*Enterococcus faecalis*	*Enterococcus faecalis*
	***Streptococcus* spp.**	*Enterococcus faecium*
Case 5	*Staphylococcus aureus*	*Staphylococcus aureus*
	***Streptococcus* spp.**	Negative
Case 6	***Enterococcus* spp.**	Negative
Case 7	***Enterococcus faecalis***	*Enterococcus faecium*
	*Pseudomonas aeruginosa*	*Pseudomonas aeruginosa*
Total	**7**	

False-positive	Cultures	BCID2
Case 1	*E. coli*	*E. coli*
		***Bacteroides fragilis***
		***Proteus* spp.**
Case 2	*Enterococcus faecalis*	*Enterococcus faecalis*
	*Streptococcus* spp.	***Enterococcus faecium***
Case 3	*Enterococcus faecalis*	***Enterococcus faecium***
	*Pseudomonas aeruginosa*	*Pseudomonas aeruginosa*
Case 4	*E. coli*	*E. coli*
	*Enterococcus casseliflavus*	***Enterococcus faecium***
		***Klebsiella pneumoniae***
Case 5	*E. coli*	*E. coli*
	*Aeromonas* spp.	***Klebsiella oxytoca***
Case 6	*E. coli*	*E. coli*
		***Enterococcus faecium***
		***Klebsiella pneumoniae***
Case 7	*E. coli*	*E. coli*
		***Klebsiella pneumoniae***
Total		10*

*The false positives included the nine bacterial species shown in this table, plus one CTX-M–producing strain, totaling ten species.

Bold terms indicate bacterial species that showed false-negative or false-positive results.

### 3.2 Comparison of BCID2 panel results with conventional culture and diagnostic performance indices

In the present study, among 77 cases, 34 were positive by bacterial culture. A comparison of the bacterial culture results with the BCID2 panel findings is shown in Table 4. Among the 34 bile culture–positive cases, the BCID2 panel identified at least one bacterial species in 28 (82.4%). In contrast, no bacteria were detected by the BCID2 panel in any of the 43 culture-negative cases. Accordingly, the diagnostic performance of the BCID2 panel for detecting at least one bacterial species in bile was as follows: positive percent agreement (PPA, equivalent to sensitivity) = 82.4%, negative percent agreement (NPA, equivalent to specificity) = 100%, positive predictive value (PPV) = 100%, negative predictive value (NPV) = 91.5%, and overall accuracy = 92.2%.

**Table 4:** Comparison of BCID2 panel results with conventional culture and diagnostic performance indices.

BCID2 vs. bile culture	Positive bile culture	Negative bile culture
BCID2 detected	28	0
BCID2 not detected	6	43

Positive percent agreement (PPA) = 28/(28 + 6) = 82.4%. Negative percent agreement (NPA) = 43/(43 + 0) = 100%. Positive predictive value (PPV) = 28/(28 + 0) = 100%. Negative predictive value (NPV) = 43/(43 + 6) = 91.5%. Accuracy = (28 + 43)/77 = 92.2%.

With respect to infection type, among the 34 bile culture–positive cases, 17 patients (50%) had monomicrobial infections and 17 patients (50%) had polymicrobial infections (Table 5). The BCID2 panel successfully detected bacteria in 12 of 17 monomicrobial infections (70.6%) and in 16 of 17 polymicrobial infections (94.1%). The detection rate was significantly higher in the polymicrobial group than in the monomicrobial group (*p* = 0.178).

**Table 5:** Comparison of BCID2 panel results between monomicrobial and polymicrobial infection.

Type of infection	BCID2 detected	BCID2 not detected	*p* value
Monomicrobial infection	12	5	0.178
Polymicrobial infection	16	1	

Fisher’s exact test.

### 3.3 Relationship between bacterial load and BCID2 panel detection among on-panel isolates

For the 45 bacterial isolates detected in bile culture, multiplex PCR system results were analyzed in relation to bacterial load, categorized as >10^6^ or <10^6^ CFU/mL (Table 6). The BCID2 panel identified 22 of 24 (91.7%) bacterial species with a load >10^6^ CFU/mL and 17 of 22 (77.3%) species with a load <10^6^ CFU/mL. Detection rates were higher for species with higher bacterial loads; however, this difference was not statistically significant (*p* = 0.344).

**Table 6:** Relationship between bacterial load and BCID2 panel detection among on-panel isolates.

Bacterial load	BCID2 panel detected	BCID2 panel not detected	*p* value
> 10^6^	22	2	0.344
< 10^6^	17	5	

Fisher’s exact test.

### 3.4 Patient characteristics according to BCID2 panel detection

The comparison of patient characteristics according to the BCID2 panel results is shown in Table 7. Patients with BCID2-positive results were significantly older and had higher Charlson comorbidity index scores, WBC counts, and CRP levels compared with those with BCID2-negative results.

**Table 7:** Patient characteristics according to BCID2 panel detection.

Factor	BCID2 detected (*n* = 28)	BCID2 not detected (*n* = 49)	*p*
Sex (M/F)	17/11	36 / 13	0.309
Age (years)	74 (34–94)	62 (22–93)	**0.004**
ASA-PS	2 (1–4)	2 (1–4)	0.198
CCI	5 (0–12)	3 (0–11)	**0.003**
Body Temperature (°C)	36.8 (36.1–38.3)	36.6 (35.4–38.2)	0.052
WBC (×10^3^/μL)	12.1 (5.1–21.8)	9.5 (2.9–21.9)	**0.016**
CRP (mg/dL)	16.14 (0.14–43.51)	5.85 (0.20–43.45)	**0.001**
T-Bil (mg/dL)	1.3 (0.3–7.4)	1.2 (0.2–4.6)	0.419

ASA-PS: American society of anesthesiologists – physical status. CCI: Charlson comorbidity index, WBC: White blood cell, CRP: C-reactive protein.

Bold values indicate statistical significance (*p* < 0.05).

## 4 Discussion

Early diagnosis and appropriate antimicrobial therapy are essential for optimizing patient outcomes in acute biliary infections. The TG18 guidelines underscore the necessity of selecting appropriate antibiotics and de-escalating from empiric broad-spectrum therapy to pathogen-directed treatment once the causative pathogen is identified (Gomi et al., 2018). Nevertheless, the increasing prevalence of antimicrobial-resistant organisms in community-acquired infections presents growing challenges for the selection of effective empiric regimens (Jean et al., 2014; Nishiwaki et al., 2025).

In our analysis, the BCID2 panel demonstrated good overall diagnostic performance in identifying causative bacteria from bile specimens. Specifically, the PPA was 82.4%, the NPA was 100%, the PPV was 100%, and NPV was 91.5%, resulting in an overall accuracy of 92.2%. The detection rate was significantly higher in polymicrobial infections than in monomicrobial infections (94.1% vs. 70.6%, *p* = 0.178). With respect to bacterial load, the BCID2 panel detected 91.7% of isolates with >10^6^ CFU/mL and 77.3% of isolates with <10^6^ CFU/mL, although this difference was not statistically significant (*p* = 0.178). Furthermore, patients with BCID2-positive results were significantly older and had higher Charlson comorbidity index scores, WBC counts, and CRP levels than those with BCID2-negative results. These findings indicate that the BCID2 panel can provide rapid and reliable pathogen detection even in samples with low bacterial loads or polymicrobial infections, and that bacterial DNA is more likely to be detected in patients with stronger inflammatory responses. The emergence of antimicrobial-resistant organisms, particularly ESBL-producing Enterobacterales, has become a significant clinical concern in acute abdominal infections. In our study, two cases of ESBL-producing *E. coli* were identified, both of which were correctly detected as CTX-M–positive by the BCID2 panel, underscoring its clinical utility.

We previously conducted a similar analysis using the Verigene system for rapid pathogen identification in bile from patients with acute cholecystitis (Watanabe et al., 2021). In that study, the bacterial identification rate in culture-positive bile samples was 35.7%. Notably, the detection rate was lower in specimens with polymicrobial specimens (26.1%) compared to those with monomicrobial samples (42.4%). Furthermore, although a higher bacterial load was associated with a significantly improved detection rate, the rate for bacteria with counts >10^6^ CFU/mL was only 58.1%. In contrast, the multiplex PCR system used in the present study demonstrated a clear improvement in detection rates under all tested conditions, enabling accurate identification even in samples with polymicrobial profiles and in bacteria with low bacterial loads. One likely factor is the difference in detection methodology: Verigene uses a microarray hybridization technique to identify bacteria, whereas the multiplex PCR system employs multiplex real-time PCR amplification. Because PCR amplification allows detection even in low bacterial loads, the multiplex PCR system successfully identified organisms that Verigene missed in low-colony-count samples.

There have been multiple reports on the usefulness of multiplex PCR systems for the rapid identification of causative microorganisms. In particular, the use of the BCID panel, originally designed for blood specimens, has been reported to improve the rate of appropriate antibiotic use and to increase the rate of successful early antibiotic de-escalation in septic patients with bloodstream infections (Britt et al., 2023; Pérez-Lazo et al., 2023). On the other hand, a limitation of multiplex PCR systems is the lack of panels specifically designed for ascitic fluid or bile in acute intra-abdominal infections, although some studies have applied the BCID panel in such contexts. Micó et al. (2015) analyzed a range of clinical specimens, including ascitic fluid and purulent fluid, using the BCID panel, and reported an overall sensitivity of 71% and a specificity of 97%. They further noted that the detection rate was 89% in specimens with a high bacterial load, such as purulent fluid, but only 25% in those with a low bacterial load, such as ascitic fluid. In our previous analysis of 10 specimens from patients with perforated peritonitis or intra-abdominal abscesses, the BCID panel achieved a detection rate of 90.5%, and combined metagenomic analysis demonstrated that bacterial genomes were detectable with as few as 19 sequencing reads (Kakizaki et al., 2023). Chayed et al. (2024), using the same BCID2 panel as in the present study, analyzed ascitic fluid from patients with acute appendicitis and reported an agreement rate with bacterial culture results of 71.4%. Although no prior study has specifically aggregated analyses of bile specimens using BCID2 panel, acute biliary infection is generally associated with a high bacterial load, suggesting that such analyses could be promising.

In acute cholangitis, the reported detection rates of major causative pathogens are 31–44% for *Escherichia coli*, 9–20% for *Klebsiella* spp., 0.5–19% for *Pseudomonas* spp., and 5–9% for *Enterobacter* spp. among Gram-negative bacteria, and 3–34% for *Enterococcus* spp. and 2–10% for *Streptococcus* spp. among Gram-positive bacteria (Gomi et al., 2018). Because these organisms are included in the BCID2 panel, we adopted this panel for the present evaluation of acute cholangitis. In contrast, the reported detection rate of anaerobes ranges from 4 to 20% (Gomi et al., 2018). In our analysis, among the total of 57 isolated bacterial species, six species (10.5%) were anaerobes, including of two isolates of *Clostridium perfringens* and two isolates of *Bacteroides vulgatus*. However, these anaerobes are not included in the BCID2 panel. Previous studies analyzing bloodstream infections not detected by BCID2 have reported that non-*fragilis Bacteroides* accounted for 12% and *Clostridium* species for 5.8% of cases (Berger et al., 2025). Careful attention is therefore required regarding the potential omission of these anaerobes, and the addition of broad-range targets capable of detecting *Bacteroides* and *Clostridium* species has been proposed for future iterations of the panel.

In recent years, the spread of ESBL-producing Enterobacterales has been recognized as a major clinical concern. Previous epidemiological studies have reported variable prevalence rates across different intra-abdominal infections. For example, Jean et al. (2014) found that 16.2% of community-acquired complicated intra-abdominal infections involved ESBL-producing Enterobacterales, with *E. coli* accounting for 73.3% of isolates. In acute cholangitis, Goo et al. (2012) reported a prevalence of 14.8%, whereas Nishiwaki et al. (2025) observed a lower rate of 3.1%. Coccolini et al. (2015) identified ESBL-producing Enterobacterales in 7.8% of acute cholecystitis cases and additionally reported KPC-producing *Klebsiella pneumoniae* strains in community-onset infections. In our present analysis, ESBL-producing *E. coli* were identified in two cases (3.5%), and in both, the CTX-M gene was correctly detected. Although this was a retrospective single-center study, our findings suggest that future prospective investigations could clarify the role of this approach in enabling early diagnosis and facilitating timely, targeted antimicrobial therapy.

In recent years, multiplex PCR–based rapid diagnostic platforms have become available for clinical use, enabling simultaneous detection of multiple pathogens and resistance genes within a short turnaround time. Comparative evaluations performed in bloodstream infections have shown that the FilmArray platform performs at least comparably to other rapid systems while delivering faster results than culture-based workflows. In a head-to-head study, FilmArray BCID showed higher sensitivity and specificity for Gram-positive organisms than GenMark ePlex (98.9 and 100% vs. 94.7 and 90.7%), with comparable performance for Gram-negatives (Oberhettinger et al., 2020). In another study, FilmArray BCID2 provided earlier pathogen identification and high specificity compared with the culture-based VITEK 2 system, while also detecting resistance genes that VITEK 2 identifies only after overnight incubation (El Sherif et al., 2022). Although these comparisons were conducted in bloodstream infections and no studies to date have compared multiplex PCR systems in intra-abdominal infection specimens, our present work (together with our previous study) extends the clinical relevance of FilmArray to bile and peritoneal samples, for which published data remain scarce (Kakizaki et al., 2023).

When using a rapid pathogen identification system, it is desirable that the occurrence of false-negatives and false-positives be kept as low as possible. In the present analysis, we identified seven false negatives. According to previous reports, the true false-negative rate for organisms included in the panel is approximately 2–3, and 67% of those false-negative cases were attributed to *Candida* species (Berger et al., 2025). These findings suggest that, while the overall false-negative rate of BCID2 panel remains low, certain bacterial groups such as *Streptococcus* and *Enterococcus* may also be at risk of under detection, highlighting the importance of cautious interpretation of negative results and the continued need for conventional culture confirmation.

In contrast, we also observed 10 false-positives in the present study. According to reports, false-positives are considered rare, and most cases have been related to the detection of coagulase-negative staphylococci, such as *Staphylococcus epidermidis* (Peri et al., 2022). These findings have been attributed to minor contamination in the sample or the detection of DNA fragments derived from non-viable organisms. In our previous study, two specimens showed false-positive results for *Streptococcus* spp.; however, metagenomic analysis was able to identify this organism in those samples (Kakizaki et al., 2023). We plan to further accumulate cases in the future and analyze trends in both false-negatives and false-positives. Moving forward, we plan to accumulate additional cases to further clarify the patterns and underlying mechanisms of both false-negatives and false-positives. Since metagenomic analysis was not performed in the present study, a comprehensive assessment of the bacterial composition in the specimens was not possible, and the reasons underlying these discrepancies remain unclear. In future investigations, for specimens showing discrepancies between bacterial culture results and the BCID2 panel, we will consider combining metagenomic analysis to achieve a more comprehensive evaluation.

In our present analysis, patients who tested positive with the BCID2 panel were significantly older and exhibited significantly higher levels of inflammatory markers. This method provides a valuable approach for rapid pathogen identification and may be especially beneficial for elderly patients with severe biliary infections, who are predisposed to worse clinical outcomes. However, this study has several limitations. First, this was a retrospective, single-center study with a relatively small sample size of 77 consecutive cases, and no formal sample size calculation was performed. Based on the results of this study, we intend to conduct a prospective multicenter study in the future. Second, as we did not prospectively evaluate the BCID2 panel in this study, the clinical impact of rapid pathogen identification has yet to be determined. Third, another limitation of the present study is that ATCC reference strains were not used to validate the assay performance. Future studies incorporating both clinical specimens and standardized ATCC control strains will be necessary to confirm the reproducibility and reliability of the BCID2 panel.

## 5 Conclusion

In this study, we achieved rapid identification of causative bacteria in bile specimens from patients with acute cholecystitis using the BCID2 panel. This method demonstrated favorable diagnostic performance and may contribute to appropriate antimicrobial stewardship, particularly amid the growing challenge of antimicrobial resistance in acute biliary infections.

## Data Availability

The raw data supporting the conclusions of this article will be made available by the authors, without undue reservation.
